# Investigating the effect of zeaxanthin on olanzapine-induced metabolic disorders in rats

**DOI:** 10.22038/AJP.2024.24352

**Published:** 2024

**Authors:** Anahita Salehsari, Mahboobeh Ghasemzadeh Rahbardar, Bibi Marjan Razavi, Hossein Hosseinzadeh

**Affiliations:** 1 *School of Pharmacy, Mashhad University of Medical Sciences, Mashhad, Iran*; 2 *Pharmaceutical Research Center, Pharmaceutical Technology Institute, Mashhad University of Medical Sciences, Mashhad, Iran*; 3 *Targeted Drug Delivery Research Center, Pharmaceutical Technology Institute, Mashhad University of Medical Sciences, Mashhad, Iran*; 4 *Department of Pharmacodynamics and Toxicology, School of Pharmacy, Mashhad University of Medical Sciences, Mashhad, Iran*

**Keywords:** Olanzapine, Zeaxanthin, Blood glucose, Metabolic syndrome, Blood pressure, Metformin

## Abstract

**Objective:**

Olanzapine (OLZ) is used by some patients with bipolar disorder and schizophrenia. Some of its side effects include weight gain and metabolic syndrome. Zeaxanthin (Zx), a yellow pigment found in egg yolk as well as some yellow and orange plants and fruits, is an anti-obesity factor that aids in the treatment of metabolic disorders. The effects of Zx on metabolic disorders caused by OLZ were investigated in this study.

**Materials and Methods:**

Female Wistar rats were randomly divided into seven groups (n=6): 1. control (vehicle); 2. OLZ (5 mg/kg, 14 days, intraperitoneal (i.p.)); 3-5. OLZ + Zx (12.5, 25, and 50 mg/kg, 14 days, gavage); 6. OLZ+ metformin (100 mg/kg, 14 days, i.p.); and 7. Zx (50 mg/kg, 14 days). Weight changes were checked every 3 days and food intake was monitored every day. Systolic blood pressure, insulin, blood sugar, triglyceride, cholesterol, high-density lipoprotein (HDL), low-density lipoprotein (LDL) and leptin levels were evaluated on the last day of the study.

**Results:**

OLZ caused an increase in weight (84.5% increase on day 15), food intake, blood pressure, triglyceride, LDL, insulin, blood sugar, and leptin compared to the control group (p<0.01 and p<0.001). The use of different doses of Zx (12.5, 25, and 50 mg/kg) and metformin decreased weight (the percentages of weigh reduction on day 15 were 91.92% for Zx (50 mg/kg) and 59.39% for metformin), food intake, systolic blood pressure, triglyceride, LDL, insulin, blood sugar, and leptin compared to the OLZ group. The amounts of cholesterol and HDL were not different in different groups.

**Conclusion:**

Zx alleviates metabolic abnormalities including hypertension, hyperglycemia, and dyslipidemia in rats.

## Introduction

Metabolic syndrome is defined as a metabolic disorder characterized by excessive blood glucose levels, central obesity, insulin resistance, dyslipidemia, overweight, and increased blood pressure which rises the incidence of cardiovascular risk factors and type II diabetes mellitus (Jalali and Ghasemzadeh Rahbardar, 2022). Obesity and overweight are now considered a growing hazard to global health. They are regarded as the primary contributors to high blood pressure, stroke, coronary heart disease, non-insulin-dependent (type II) diabetes, and several malignancies (Razavi et al., 2020; Sanati et al., 2018). Although second-generation antipsychotic medications, including olanzapine (OLZ), are frequently used to treat schizophrenia, bipolar disorder, and other mental illnesses, they promote obesity and other dangerous metabolic conditions, such as dyslipidemia (Buhagiar and Jabbar, 2019; Su et al., 2020). A growing body of research indicated that second-generation antipsychotics could negatively affect lipid metabolisms which could ultimately result in type II diabetes, insulin resistance, and glucose intolerance (Chen et al., 2017; Vantaggiato et al., 2019). Additionally, long-term use of atypical antipsychotic medications such as OLZ, can increase serum leptin levels and cause metabolic abnormalities like diabetes, hyper-insulinemia, insulin resistance, dyslipidemia, and hypertension (Ardakanian et al., 2022; Razavi et al., 2017). Therefore, it is essential to comprehend the molecular mechanisms underlying the dysregulation of lipid metabolisms caused by antipsychotics to create efficient preventative and treatment plans for these negative side effects (Rojo et al., 2015). The mechanisms underlying antipsychotic-induced weight gain and metabolic disorders include blocking the dopamine (D_2_), histamine (H_1_), muscarinic (M_3_), and 5-hydroxytryptamine (serotonin) 2C (5-HT2C) receptors, as well as the 5-hydroxytryptamine receptor (HTR)2C and HTR2A gene polymorphisms, a marked increase in food intake and appetite, insulin resistance, and a decrease in physical activity (Lian et al., 2014; Liu et al., 2017b; Mota de Sá et al., 2017; Xu and Zhuang, 2019).

Phytochemicals with antioxidant properties are being studied extensively in nutrition for the prevention and treatment of a variety of diseases including metabolic disorders, diabetes, obesity, and cardiovascular disease (Eisvand et al., 2022; Fadishei et al., 2021; Galavi et al., 2021; Rahbardar et al., 2022).

Zeaxanthin (Zx) is a member of the carotenoids known as the xanthophyll family. In contrast to certain other carotenoids, this substance has a stronger polar orientation, antioxidant activity, and esterification potential due to the presence of ketones in the ionone rings and a terminal hydroxyl. By squelching free radicals and singlet oxygen, carotenoids function as antioxidants. This lipophilic pigment, like other carotenoids, cannot be produced by the body and must be obtained through diet. Zx is responsible for the yellow tone of animal fat, egg yolks, and the macula of the human retina. Zx is said to be present in foods including fried egg yolks, chives, oranges, peppers, and yellow corn flour (López-Cervantes and Sánchez-Machado, 2019; Zhang et al., 2021). The oral administration of Zx to obese mice improved dyslipidemia and slowed obesity progression (Liu et al., 2017a). Moreover, intake of lutein/Zx was previously found to be positively connected with serum high-density lipoprotein (HDL) cholesterol but adversely correlated with serum low-density lipoprotein (LDL) cholesterol and serum triglyceride levels (Blesso et al., 2013; Xu et al., 2013). According to certain research, adults' risk of having an increased waist circumference, hypertriglyceridemia, and hypertension is inversely correlated with blood lutein/Zx concentration (Beydoun et al., 2011; Sugiura et al., 2008). 

Therefore, this study assessed the effects of Zx (12.5, 25, and 50 mg/kg, 14 days, gavage) on metabolic disorders induced by OLZ (5 mg/kg, 14 days, intraperitoneal (i.p.)) in female rats because many schizophrenic patients receiving OLZ are at greater risk of weight gain besides further side effects, and Zx is recognized to be efficacious in controlling and managing these metabolic disorders.

## Materials and Methods

### Chemicals

OLZ was purchased from Sobhan, Iran. Zx was attained from Tinab Shimi, Iran. Ketamine 10% and xylazine 2% were prepared from Alfasan, Iran. Acetic acid was bought from Merck, Germany.

### Animals

In this investigation, female Wistar rats weighing on average 220 g were used. The rats were obtained from the Mashhad School of Pharmacy's animal breeding center, and each two rats were housed in separate cages with free access to water and 50 g of food each day, at a temperature between 22 and 25°C, with a 12/12-hr light/dark cycle. However, since they needed to be fasted to analyze variables like blood sugar and blood lipids, the animals' access to food was restricted on the final night of the trial for 12 hr before blood sampling. Additionally, all animal tests were carried out by the guidelines established by the Mashhad School of Pharmacy ethics committee (code 958932) (IR.MUMS.PHARMACY.REC.1400.020). These guidelines are also consistent with the Internationally Recognized Principles for Animal Use and Care (Zimmermann, 1983).

### Study groups

The experiments were performed on 42 female Wistar rats, which were divided into 7 groups of 6.

1: Animals receiving OLZ solvent (normal saline and 50 µL citric acid, 14 days, i.p.) and Zx solvent (corn oil without antioxidants, gavage); 2: Animals receiving a dose of 5 mg/kg of OLZ, 14 days, i.p. (Ardakanian et al., 2022); 3: Animals receiving OLZ+ Zx 12.5 mg/kg, 14 days, gavage (Chamberlain et al., 2009); 4: Animals receiving OLZ+ Zx 25 mg/kg, 14 days, gavage (Chamberlain et al., 2009); 5: Animals receiving OLZ+ 50 mg/kg Zx, 14 days, gavage (El-Akabawy and El-Sherif, 2019); 6: Animals receiving OLZ+ 100 mg/kg metformin, 14 days, i.p. (Akhtar et al., 2007); and 7: Animals receiving a dose of 50 mg/kg Zx, 14 days, gavage.

### Measuring food consumption and weight changes

Throughout the study, food consumption was monitored daily, and body weight was measured every three days. Two female rats weighing approximately 220 g were kept in separate cages, and each rat was given 25 g of food, with a total of 50 g of food placed in the cage. After 24 hr, the amount of food left was calculated, and the amount consumed was estimated. 

### Systolic blood pressure measurement

The rat tail artery and a non-invasive tail-cuff method (Harvard Apparatus, MLT125R) were used to measure systolic blood pressure. The animal was first anesthetized using an i.p. injection of 60 mg/kg ketamine and 6 mg/kg xylazine. The rat's tail was then cleaned and warmed by lamplight. By pressing the corresponding key on the blood pressure measuring device (NIBP Controller), air enters the blood pressure cuff and after a few seconds, the heart rate returns to normal. Systolic blood pressure is defined as the point at which the animal's heart rate and pulse amplitude return to normal. Systolic blood pressure was measured 5 times and finally, the average pressure is reported as the final systolic blood pressure (Ardakanian et al., 2022; Fadishei et al., 2021).

### Measuring the serum levels of fast blood sugar (FBS), insulin, triglyceride, cholesterol, HDL, and LDL

The animals were sacrificed by decapitation after the study period, and the serum was sent to the laboratory to measure the levels of insulin, glucose, triglycerides, cholesterol, HDL, and LDL.

### Measuring serum level of leptin

The amount of leptin in blood serum samples was determined using an enzyme-linked immunosorbent assay (ELISA) kit (Shanghai Crystal Day Biotech) that was based on biotin double antibody sandwich technology. The leptin kit works based on the unique relationship that exists between antibodies and leptin. The wells were filled with a blank solution, various standard dilutions, and samples from various groups. Leptin in the samples binds to the antibody attached to the plate wall after 60 min of incubation at 37°C. After that, the wells were washed five times with the washing solution, each time for 30 sec. Following that, chromogen solutions were added to the wells and incubated for another 10 min. Then, a yellow color was visible, which turned blue when the final solution was added. Finally, the ELISA reader was used to measure color intensity at 450 nm, and a standard graph was used to compute and report the samples' leptin content (Ardakanian et al., 2022).

### Statistical analysis

The statistical calculations were done using the Prism 9 program. Results are shown as Mean±standard deviation (SD). One-way and two-way ANOVA, as well as Tukey-Kramer and Bonferroni post-tests, were employed to compare the various groups. Two-way ANOVA was applied to compare the average body weight gain and average food intake. The definition of a significant difference was a p-value of less than 0.05.

## Results

### Effect of Zx and OLZ on weight changes

The rats of all groups were weighed on days 0, 3, 6, 9, 12, and 15 and their weight changes were compared to their weight on day 0.

**Figure 1:**
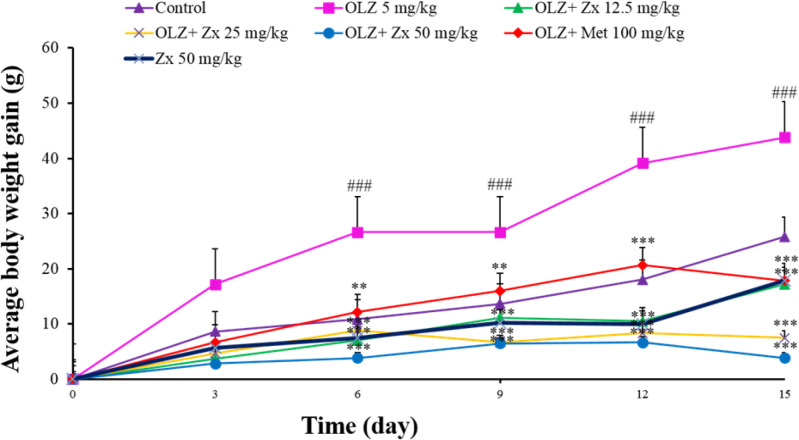
The effect of OLZ and Zx on the weight changes of rats. OLZ (5 mg/kg, 14 days, i.p.), Zx (50, 25, 12.5 mg/kg, 14 days, gavage), and metformin (100 mg/kg, 14 days, i.p.) were administered to the animals. Data are Mean±SD (n=6). Two way-ANOVA test and Tukey-Kramer post-test were used to check the statistical difference. ###p<0.001 compared to control, ***p<0.001 and **p<0.01 compared to OLZ 5 mg/kg. OLZ: olanzapine, Zx: zeaxanthin.

The results of the weight change of rats in different groups show that the administration of OLZ (5 mg/kg) caused an increase in weight (84.5% increase on day 15) compared to the control group on days 6-15 (p<0.001). Also, the administration of Zx with different doses of 12.5 mg/kg (39.15% decrease on day 15), 25 mg/kg (82.89% decrease on day 15), and 50 mg/kg (91.92% decrease on day 15) along with the injection of OLZ for 14 days was able to reduce the weight of rats from day 6-15 (p<0.001). Furthermore, it can be seen that the injection of metformin (100 mg/kg) along with OLZ on days 6-9 (p<0.01) and 12-15 (p<0.001) (59.39% decrease on day 15) caused weight loss compared to the control group. Zx (50 mg/kg) did not change the weight of rats compared to the control group (Figure 1) (Table 1).

### Effect of Zx and OLZ on average food intake

**Figure 2:**
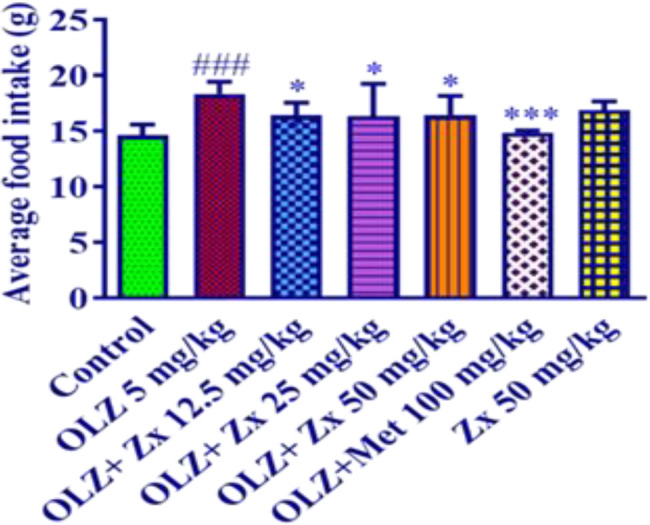
The effect of OLZ and Zx on average food intake. OLZ (5 mg/kg, 14 days, i.p.), Zx (50, 25, and 12.5 mg/kg, 14 days, gavage), and metformin (100 mg/kg, 14 days, i.p.) were administered to the animals. Data are Mean±SD (n=6). One way-ANOVA test and Tukey-Kramer post-test were used to check the statistical difference. ###p<0.001 compared to control, ***p<0.001 and **p<0.01 compared to OLZ 5 mg/kg. OLZ: olanzapine, Zx: zeaxanthin.

The average amount of food consumed in the samples of the OLZ group (5 mg/kg) increased compared to the control group (p<0.001). Also, administration of Zx with doses of 12.5, 25, and 50 mg/kg along with OLZ injection for 14 days could significantly decrease food intake compared to the OLZ group (p<0.05). Administration of metformin (100 mg/kg) along with OLZ also attenuated the average amount of food consumed in comparison with the control group (p<0.001). Furthermore, Zx (50 mg/kg) did not change the average food intake compared to the control group (Figure 2). 

### Effect of Zx and OLZ on systolic blood pressure

Administration of OLZ (5 mg/kg) caused a significant increase in systolic blood pressure compared to the control group (p<0.001). The administration of Zx (12.5 and 25 mg/kg) (p<0.01) and (50 mg/kg) (p<0.001) together with OLZ were effective in reducing the systolic blood pressure compared to the OLZ group. 

**Figure 3:**
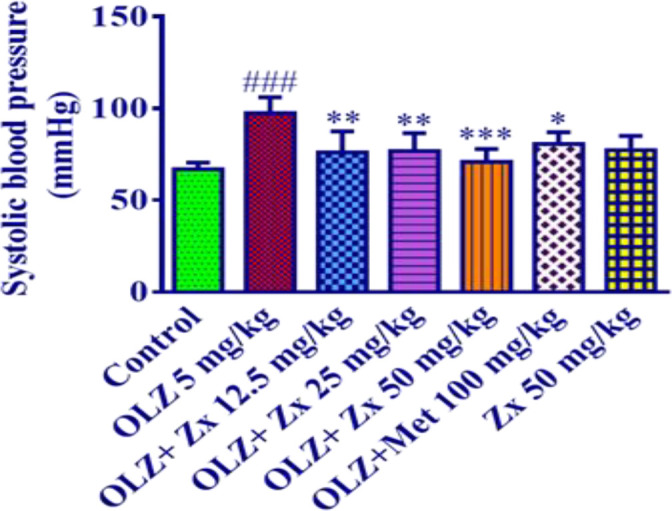
The effect of OLZ and Zx on systolic blood pressure. OLZ (5 mg/kg, 14 days, i.p.), Zx (50, 25, and 12.5 mg/kg, 14 days, gavage), and metformin (100 mg/kg, 14 days, i.p.) were administered to the animals. Data are Mean±SD (n=6). One way-ANOVA test and Tukey-Kramer post-test were used to check the statistical difference. ###p<0.001 compared to control, ***p<0.001, **p<0.01 and *p<0.05 compared to OLZ 5 mg/kg. OLZ: olanzapine, Zx: zeaxanthin.

Metformin injection (100 mg/kg) along with OLZ injection was able to significantly reduce systolic blood pressure compared to the OLZ group (p<0.05). Furthermore, Zx (50 mg/kg) alone did not change the mean systolic blood pressure compared to the control group (Figure 3). 

### Effect of Zx and OLZ on serum insulin levels

The amount of insulin in the samples of the OLZ group (5 mg/kg) increased compared to the control group (p<0.01). Also, administration of Zx with doses (12.5 and 50 mg/kg) along with OLZ injection for 14 days could cause a significant change compared to the OLZ group (p<0.01), but Zx (25 mg/kg) along with OLZ could not cause a significant change in the amount of blood insulin compared to the OLZ group. Administering metformin (100 mg/kg) along with OLZ also caused a significant change in blood insulin levels (p<0.05). Zx (50 mg/kg) did not change the blood serum insulin level compared to the control group (Table 2).

### Effect of Zx and OLZ on serum FBS level and lipid profile

The results show that the administration of OLZ (5 mg/kg) caused a significant increase in blood FBS compared to the control group (p<0.001). The administration of Zx (12.5, 25, and 50 mg/kg) along with OLZ was effective in reducing FBS compared to the OLZ group (p<0.05). Injection of metformin (100 mg/kg) along with injection of OLZ could significantly decrease the amount of FBS in the blood (p<0.01). Zx (100 mg/kg) alone did not change the amount of blood FBS compared to the control group (Table 2). The amount of cholesterol in the samples of the OLZ group (5 mg/kg) increased compared to the control group, but this increase was not significant. Moreover, Zx (50 mg/kg) did not change the blood serum cholesterol level compared to the control group.

The amount of triglycerides in the samples of the OLZ group (5 mg/kg) increased compared to the control group (p<0.001). Also, administration of Zx with different doses (12.5, 25, and 50 mg/kg) along with OLZ injection for 14 days, caused a significant change compared to the OLZ group (p<0.001). The administration of metformin (100 mg/kg) along with OLZ also caused a significant reduction in the amount of blood triglyceride (p<0.001). Furthermore, Zx (50 mg/kg) did not change the blood serum triglyceride level compared to the control group. 

As can be seen in Table 2, the administration of OLZ (5 mg/kg) did not cause any changes in HDL blood levels compared to the control group. Besides, Zx (50 mg/kg) did not change the level of HDL in blood serum compared to the control group. 

The administration of OLZ (5 mg/kg) caused a significant increase in serum LDL level compared to the control group (p<0.01). Also, the administration of Zx (12.5 mg/kg) (p<0.05) along with the injection of OLZ significantly reduced the amount of LDL in blood serum. But, Zx (25 and 50 mg/kg) could not cause significant changes in blood LDL levels compared to the OLZ group. Administering metformin along with OLZ also caused a significant decrease in blood LDL levels compared to the OLZ group. Moreover, the injection of Zx (50 mg/kg) did not change the level of blood serum LDL compared to the control group.

### The effect of Zx and OLZ on serum leptin levels

The administration of OLZ (5 mg/kg) caused a significant increase in leptin levels in the blood serum compared to the samples of the control group (p<0.001). 

**Figure 4:**
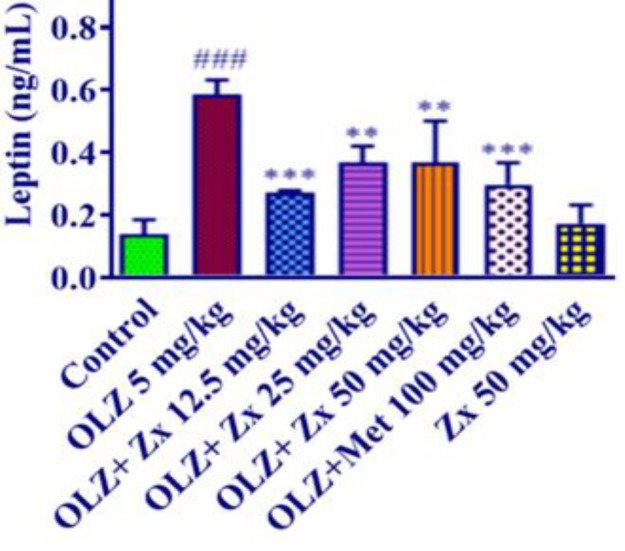
The effect of OLZ and Zx on blood leptin levels. OLZ (5 mg/kg, 14 days, i.p.), Zx (50, 25, and 12.5 mg/kg, 14 days, gavage), and metformin (100 mg/kg, 14 days, i.p.) were administered to the animals. Data are Mean±SD (n=6). One way-ANOVA test and Tukey-Kramer post-test were used to check the statistical difference. ###p<0.001 compared to control, and ***p<0.001 and **p<0.01 compared to OLZ 5 mg/kg. OLZ: olanzapine, Zx: zeaxanthin.

Also, administration of Zx (12.5 mg/kg) (p<0.01), Zx (25 and 50 mg/kg) (p<0.01) along with OLZ injection caused a significant decrease in serum leptin level compared to the OLZ group. Furthermore, the injection of metformin (100 mg/kg) along with OLZ could significantly reduce the leptin level compared to the OLZ group (p<0.001). Furthermore, Zx (50 mg/kg) administration did not change the leptin level compared to the control group (Figure 4).

## Discussion

The effect of Zx on weight gain and metabolic disorders induced by OLZ was examined in the current study. As compared to the control group, the results showed that intraperitoneal injection of OLZ (5 mg/kg, 14 days) to rats increased their weight, food intake, blood pressure, triglycerides, LDL, blood sugar, insulin, and leptin levels. In comparison to the OLZ group, the combination of different dosages of Zx (12.5, 25, and 50 mg/kg, 14 days, gavage) or metformin (100 mg/kg, 14 days, i.p.) along with OLZ reduced weight, food intake, systolic blood pressure, triglyceride, LDL, blood sugar, insulin, and leptin. There were no differences in the levels of HDL or cholesterol between the groups.

Eighty percent of patients taking OLZ at the beginning of their treatment for psychosis face an increase of ≥7% in their body weight (Huang et al., 2020). When assessing either short- or long-term anthropometric outcomes, OLZ is invariably listed as having the greatest impact on user body weight increases, except clozapine. OLZ was found to be the antipsychotic most likely to cause clinically significant weight gain in a 2019 meta-analysis of 402 randomized controlled trials with data from 53,463 participants with multi-episode schizophrenia (Huhn et al., 2019). Antipsychotic-induced weight gain is a particularly significant side effect because it influences cardiometabolic outcomes, such as the onset of type 2 diabetes and subsequent cardiovascular disease, which accounts for about 60% of the excess mortality among people with schizophrenia (Firth et al., 2019). Such high rates of physical comorbidity not only drastically reduce life expectancy but also increase the personal, social, and financial burden of mental illness throughout the lifespan. Considerable weight gain might similarly have an impact on mental health outcomes. One of the most displeasing side effects of antipsychotic therapy is consistently listed as weight gain, which frequently leads to partial or complete antipsychotic non-adherence and future resistance to treatment participation (Fitzgerald et al., 2021; Velligan et al., 2017). Additionally, it has been suggested that metabolic syndrome and obesity may each independently predict relapse and re-hospitalization in people with severe mental illnesses (Manu et al., 2014). Another study also claimed that treatment with OLZ for schizophrenia patients frequently results in weight gain and metabolic disorders, which may be influenced by increased appetite (Huang et al., 2020). In an animal model, treatment with OLZ (1 kg of the control diet was compounded with 50 mg of OLZ) changed energy expenditure and physical activity, and increased food intake, and glucose tolerance. Furthermore, in mice lacking the serotonin 2C receptor, OLZ-induced hyperphagia and weight gain were diminished (Lord et al., 2017). The reason for using female rats is that, unlike male rats, the clinical phenotype associated with weight gain is easily created in female rats (Ersland et al., 2019). In rats, there is a significant gender difference in weight gain, with female rats being much more likely to gain weight than males. For example, 15 of 17 studies in rats chronically treated with OLZ reported weight gain in female rats, but only one of seven studies reported weight gain in male rats. It should be noted that this gender difference is not comparable to human clinical findings (Boyda et al., 2010). Another study found that OLZ caused rapid weight gain in female rats, but this weight gain was not as noticeable in male rats, which is consistent with previous reports. Both sexes of rats treated with OLZ had an increase in visceral fat, though males only at higher doses (Davey et al., 2012). Moreover, another study assessed the effect of OLZ (5 mg/kg, 14 days, i.p.) on female Wistar rats and the obtained data revealed that OLZ significantly increased the body weight and food intake compared to the control group (Ardakanian et al., 2022). In line with previous studies, our results also illustrated weight gain and enhanced food intake in animals that received OLZ compared to the control group. It is essential to note that the administration of Zx 50 mg/kg alone to rats decreased their weight in comparison to the control group, but the administration of Zx 50 mg/kg along with OLZ to rats reduced their weight more (as shown in Figure 1), which indicates that Zx acts more effectively in obese rats. On the other hand, concurrent treatment with Zx and OLZ reversed the alterations induced by OLZ. Likewise, Zx (100 mg/kg, 17 weeks, p.o.) administration appeared to be an effective way to reverse the metabolic changes brought on by a high-fat diet (increased visceral adipose tissue, weight gain) in male Wistar rats (Al-Thepyani et al., 2022). It was also indicated that Zx reduces obesity by modifying the gut microbiota and activating the β3-adrenergic receptor to promote inguinal fat thermogenesis in mice (Xie et al., 2021). Furthermore, Zx inhibits lipogenesis and reduces adipose weight, adipocyte size, and intracellular lipid content to have anti-adipogenic and anti-obesity effects in mice (Zx 20 mg/kg, 4 weeks, p.o.) and/or in 3T3-L1 adipocytes (Zx 5–15 μM) (Liu et al., 2017a).

Leptin plays a crucial role in homeostasis, controlling appetite, and regulating energy levels. It is produced and secreted by enterocytes and adipose cells (Fadishei et al., 2021; Jalali and Ghasemzadeh Rahbardar, 2022). Leptin is regarded as an indicator when taking atypical antipsychotic medications due to its correlation with changes in body weight and glucose metabolism (Ardakanian et al., 2022). Raised leptin levels synchronicity with obesity is typically interpreted as a sign of leptin resistance because leptin reduces body weight and food intake. Cellular leptin resistance which is caused by several cellular processes that are stimulated by obesity, increases the amount of weight gain induced by environmental and genetic factors (Myers et al., 2010). The fourth week of a study involving 13 schizophrenia patients receiving OLZ showed an increase in serum leptin levels (Hosojima et al., 2006). OLZ (5 mg/kg) was administered to 24 female Wistar rats throughout a 12-day study, and this increased leptin levels in comparison to the controls (Malekzadeh et al., 2019). In consistency with previous studies, intraperitoneal injection of OLZ in our research was also followed by an increased amount of leptin in rats’ serum. However, the simultaneous administration of OLZ and Zx significantly reduced leptin levels compared to the animals in the OLZ group. A former study also reported a reduced amount of leptin in obese mice fed with Zx (Xie et al., 2021). Similarly, it was revealed that Zx (100 mg/kg, 17 weeks, p.o.) resulted in lower amounts of leptin compared with the rats in the high-fat diet group (Al-Thepyani et al., 2022).

Some of the primary risk factors for obesity and metabolic disorder are dyslipidemia, hypertension and insulin resistance (Najafi et al., 2022; Yahyazadeh et al., 2021; Yarmohammadi et al., 2021). It has been observed that administration of OLZ (3 mg/kg/day, 4 weeks) to mice altered the lipid metabolism in the liver, blood lipid profile, and aortic cholesterol homeostasis, besides increasing the mean arterial pressure. It also accumulated lipids in the liver, particularly cholesterol and attenuated HDL (Chen et al., 2018). A clinical trial on 30 schizophrenic patients illustrated that OLZ augmented weight, visceral fat, and cholesterol (Salviato Balbão et al., 2014). OLZ also enhanced weight and triglyceride levels in a study of 25 patients over 12 weeks (Osser et al., 1999). Our findings also indicated that the administration of OLZ to female Wistar rats caused an increase in systolic blood pressure, triglyceride, LDL, insulin, and blood sugar. But it did not affect HDL and cholesterol. The discrepancy seen between our study and other studies on cholesterol can be explained by animal breed, gender, and age, purity of OLZ and duration of administration. Conversely, the simultaneous administration of OLZ and Zx ameliorated the alterations induced by OLZ and reduced blood pressure, LDL, triglyceride, insulin, and blood sugar. Another research also reported that the administration of Zx (200 and 400 mg/kg, 4 weeks, p.o.) to diabetic rats significantly reduced the levels of blood glucose, cholesterol, triglyceride, and LDL, and increased the HDL levels (Kou et al., 2017). A study was carried out to assess the effect of Zx (60 mg/kg, 7 weeks, gavage) on the blood vessel damage induced by high lipid levels in a model of hyperlipidemia in quail and it was observed that serum triglyceride, total cholesterol, and LDL concentrations were significantly lower in the Zx group compared to the model group, whereas serum HDL concentrations were significantly higher (Li et al., 2011). Furthermore, a cross-sectional study claimed that a lower prevalence of hypertension is linked to greater levels of six mixed serum carotenoids including lutein/ Zx, α-carotene, trans-β-carotene, cis-β-carotene, β-cryptoxanthin, and trans-lycopene (Zhu et al., 2022). In the insulin test of the current study, it was discovered that the middle dose of Zx did not significantly reduce the amount of serum insulin, although the data range was nearly identical in all treatment groups. This data can be explained by laboratory error, a small number of data points, and animal differences. Furthermore, only the lowest dose of Zx was successful in attenuating LDL levels, while the two higher doses were unable to significantly reduce this factor, which may be because lower doses of Zx are more effective in depressing the mechanisms that cause metabolic syndrome. It is also possible that higher doses (above 50 mg/kg) of Zx are oxidants. The lack of a significant difference in cholesterol and HDL levels observed in this study experiments may be due to the requirement for longer period of OLZ administration than that of selecting for this study, and perhaps more sensitive rat kits might be required.

Zx is known to possess antioxidant properties due to its ability to scavenge free radicals and reduce oxidative stress (Murillo et al., 2019). Metabolic disorders induced by OLZ, such as hypertension, hyperglycemia, and dyslipidemia, are frequently accompanied with increased oxidative stress (Ardakanian et al., 2022). Zx may help to alleviate the oxidative damage induced by OLZ by acting as an antioxidant, therefore reducing these metabolic alterations. This mechanism may explain the observed improvements in blood pressure, blood sugar, and lipid profiles in the Zx-treated groups. Another potential mechanism to consider is the effect of Zx on serotonin receptors. OLZ is an atypical antipsychotic medication that acts as a serotonin antagonist (Del Fabro et al., 2019). It is well known that it affects serotonin signaling, which results in weight gain and metabolic syndrome (Almotayri et al., 2021). By regulating serotonin synthesis or by directly interacting with the receptors, Zx has the ability to modify serotonin receptors (El-Baz et al., 2021). Zx may help to prevent the metabolic disorders stimulated by OLZ by modulating serotonin pathways. However, further research is necessary to elucidate the exact nature of Zx interaction with serotonin receptors and its role in ameliorating OLZ-induced metabolic disorders. Furthermore, alternative pathways that may be involved in the reported effects of Zx should be considered. Zx, for example, may alter other molecular pathways implicated in metabolism, inflammation, or hormone control. Future studies should seek to investigate these various processes in order to acquire thorough knowledge of how Zx amend OLZ-induced metabolic disorders.

Metformin was used as a positive control in the current investigation because it has been used to successfully stop the transition from glucose intolerance to type 2 diabetes in adults and patients with prediabetes by reducing weight gain, hyperinsulinemia, and dyslipidemia (Bassols et al., 2019). In our study, concurrent administration of metformin (100 mg/kg, 14 days, i.p.) and OLZ could significantly reduce weight, food intake, systolic blood pressure, triglyceride, LDL, blood sugar, insulin, and leptin level in comparison with the OLZ group. It is worth mentioning that the results obtained from all doses of Zx, especially the lowest dose, can compete with the effects of metformin.

The findings of the current research demonstrate that OLZ administration rises food intake, weight gain, and blood pressure. It also increases leptin, insulin, blood sugar, triglyceride, and LDL in blood serum. Conversely, the concurrent administration of Zx and OLZ resulted in reduced food intake, weight loss, and decreased systolic blood pressure. Zx also decreased leptin, triglyceride, LDL, insulin, and blood sugar levels. It might be suggested that leptin reduction is one of the weight-reducing mechanisms of Zx.

**Table 1:** Weight change comparisons in groups (the weight change in each group is the difference in weight between various days compared to the initial day=day 0)

**Study groups**	**Day 3 **	**Day 6 **	**Day 9 **	**Day 12 **	**Day 15**
OLZ (5 mg/kg)	NS	###	###	###	###
Zx (12.5 mg/kg)+ OLZ	NS	***	***	***	***
Zx (25 mg/kg)+ OLZ	NS	***	***	***	***
Zx (50 mg/kg)+ OLZ	NS	***	***	***	***
Met (100 mg/kg)+ OLZ	NS	**	**	***	***
Zx (50 mg/kg)	NS	NS	NS	NS	NS

Two way-ANOVA test and Tukey-Kramer post-test were used to check the statistical difference. ###p<0.001 compared to control, ***p<0.001 and **p<0.01 compared to OLZ. OLZ: olanzapine, Zx: zeaxanthin, NS: non significant).

**Table 2:** The effect of OLZ and Zx on FBS and serum lipid profile.

	**Control**	**OLZ** **(5 mg/kg)**	**Zx (12.5 mg/kg)+ OLZ**	**Zx (25 mg/kg)+ OLZ**	**Zx (50 mg/kg)+ OLZ**	**Met (100 mg/kg)+ OLZ**	**Zx** **(50 mg/kg)**
Insulin (mic IU/ml)	0.11±0.00	0.27±0.03 ##	0.11875±0.01 **	0.15±0.05	0.115±0.02 **	0.12±0.03 *	0.13±0.02
FBS (mg/dl)	113.25±3.99	265.80±29.71 ###	155.66±3.93 *	154.33±12.21 *	154.00±18.03 *	127.33±13.00 **	180.5±15.65
Cholesterol (mg/dl)	39.60±2.43	42.16±2.37	41.66±2.60	41.16±1.81	46.16±3.78	44.66±3.43	38.16±3.55
TG (mg/dl)	113.16±11.55	473.20±62.80 ###	103.16±10.94 ***	107.16±10.10 ***	99.33±12.42 ***	104.16±16.90 ***	104.00±23.54
HDL (mg/dl)	42.33±2.29	43.66±2.29	46.16±3.19	47.83±2.01	51.66±3.95	45.00±3.34	41.83±2.86
LDL (mg/dl)	10.16±0.81	17.40±1.83 ##	11.00±.58 *	13.25±0.25	15.40±.90	11.00±1.55 *	12.5±0.86

FBS: Fasting blood sugar, HDL: High-density lipoprotein, LDL: Low-density lipoprotein, Met: metformin, OLZ: Olanzapine, TG: Triglyceride, Zx: zeaxanthin. ###p<0.001 and ##p<0.01 compared to the control group and ***p<0.001, **p<0.01, and *p<0.05 compared to the OLZ group.
